# Carpal Tunnel Syndrome Caused by Tumoral Calcinosis

**DOI:** 10.1155/2015/170873

**Published:** 2015-07-21

**Authors:** Atsuyuki Inui, Takahiro Suzuki, Takeshi Kokubu, Ryosuke Sakata, Yutaka Mifune, Masahiro Kurosaka

**Affiliations:** Department of Orthopaedic Surgery, Kobe University Graduate School of Medicine, Kusunoki-cho, Chuo-ku, Kobe 650-0017, Japan

## Abstract

We present a case of carpal tunnel syndrome caused by systemic tumoral calcinosis. A 54-year-old woman experienced hand numbness that persisted for nine months. She had no family history or medical problem. A plain radiograph of her right wrist showed a calcified mass on the volar side of the wrist joint. The patient also experienced pain in her contralateral wrist joint and both right and left shoulders, which had calcification on radiography around the joint. Her condition was diagnosed as carpal tunnel syndrome caused by systemic tumoral calcinosis and a resection biopsy was performed. Histopathological analysis of the biopsied specimen showed basophile deposition inside the fibrous connective tissue. At 14 months after the treatment, she no longer had pain or numbness in her fingers and there was no recurrence of the mass. This patient's condition is considered as a case of nonfamilial, systemic primary tumoral calcinosis. Since incomplete resection leads to a recurrence of the lesion, a follow-up radiography examination is needed to monitor symptom recurrence.

## 1. Introduction

Carpal tunnel syndrome (CTS) is a common median nerve mononeuropathy that causes numbness and pain in the volar surface of the thumb through the radial half of the ring finger. Entrapment by the transverse carpal ligament is a common cause of CTS; however, trauma, pregnancy, space-occupying lesions, and dialysis related amyloidosis can also cause secondary CTS [1].

Tumoral calcinosis is a rare histopathological syndrome that is characterized by the deposition of calcium in periarticular areas, especially around the hip, shoulder, and elbow [2]. There are several reports of CTS caused by a solitary calcareous mass [3–5]. However, cases of CTS caused by systemic tumoral calcinosis have never been reported. Here, we report the case of CTS caused by systemic tumoral calcinosis. The patient had calcified lesions in several parts of her body.

## 2. Case Presentation

**Figure 1:**
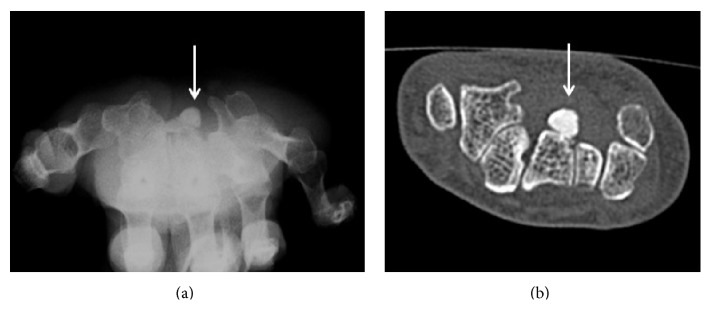
A plain radiograph showing a calcified mass on the volar surface of the wrist joint (a). A computed tomogram showing a lesion that has a high-density mass (b).

**Figure 2:**
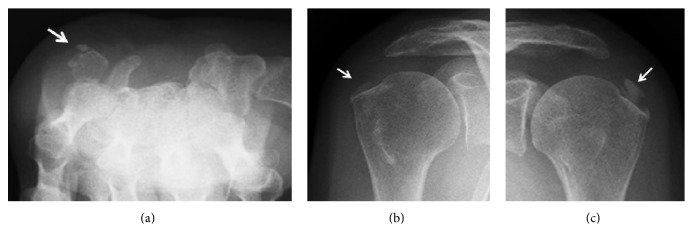
Calcification was observed on the contralateral wrist joint (a) and bilateral shoulder joints (b, c).

**Figure 3:**
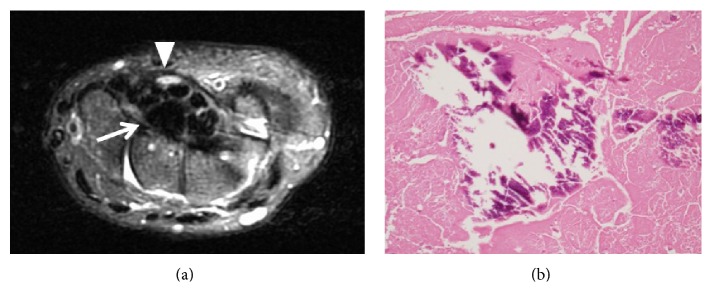
(a) T2-weighted, fat-suppressed, magnetic resonance imaging (MRI) showing the median nerve with high-signal intensity (arrowhead) and the mass inside the carpal tunnel (arrow). (b) Microscopic appearance of the excised calcified mass.

A 54-year-old woman presented to the clinic with numbness in her right thumb through her ring finger that had persisted for nine months. She had a Tinel's sign on the volar side of her wrist and weakness of her opponens pollicis muscle. An electrophysiological examination indicated distal motor latency (DML) of her median nerve as 11.0 ms and the sensory nerve conduction velocity (SCV) was not measurable. The DML of her contra lateral hand was 3.3 ms and the SCV was 55.3 m/s. At our institute, DML < 4.5 ms and SCV > 40 m/s are considered normal values; therefore the patient met the diagnosis criteria for CTS. The patient's blood concentrations of calcium, phosphate, and uric acid were 9.8 mg/dL (normal range, 8.8–10.1 mg/dL), 4.7 mg/dL (normal range, 2.4–4.5 mg/dL), and 7.3 mg/dL (normal range, 2.6–5.1 mg/dL), respectively. A plain radiograph of her right wrist showed a calcified mass on the volar side of the wrist joint (Figure 1(a)). Computed tomography showed a high-density mass in the carpal tunnel, having a volume of 2.7 × 1.2 × 1 cm^3^ in the carpal tunnel (Figure 1(b)). The patient also reported pain in her left wrist joint and both right and left shoulders, which had calcification around the joints (Figures 2(a)–2(c)). Magnetic resonance imaging (MRI) revealed a low-intensity lesion in the T1 and T2WI, and the boundary between the lesion and the surrounding tissues was clear. The fat-suppressed T2 image showed the median nerve with high-signal intensity (Figure 3(a)). The patient's condition was diagnosed with CTS caused by systemic calcified mass and a resection biopsy was performed. A 5 cm incision was made on the volar side of her wrist joint and the transverse carpal ligament was transected. The median nerve was compressed by an underlying round white mass located in the carpal tunnel. There was no adhesion to surrounding tissues. Pathological examination showed basophile deposition inside the fibrous connective tissue (Figure 3(b)). A component analysis showed the mass having 82% calcium phosphate and 17% calcium carbonate.

At 14 months after treatment, the patient had no pain or numbness in her fingers and muscle weakness of opponens pollicis had improved. Electrophysiological examination of her affected side showed a DML of 3.7 ms and SCV of 47.1 m/s, which was within normal limits.

## 3. Discussion

While the majority of CTS cases are idiopathic, some are secondary CTS caused by space-occupying lesions, tenosynovitis, vascular anomalies, or malunited distal radial fractures [6]. Electrophysiological examination is widely used to diagnose and evaluate the severity of CTS. However, not all surgeons recommend additional routine imaging examinations because of increased cost and questionable reliability [7, 8]. Generally, patients with idiopathic CTS have several common characteristics (female gender, middle age, and bilateral involvement) [9]. Consequently, unilateral CTS cases with normal electrophysiological findings in the contralateral side should have further examination to rule out a possibility of secondary CTS [7]. Ultrasound or MRI of carpal tunnel is useful in determining the cause of secondary CTS [7, 10]. Chen et al. reported space-occupying lesions in 23 out of 779 patients (2.9%) who had had received treatment for CTS [9]. Nakamichi and Tachibana reported a higher incidence of space-occupying lesions in unilateral than in bilateral CTS [6].

Several diseases that result in abnormal calcareous lesion, such as gout, pseudogout, tumoral calcinosis, or idiopathic calcification, have been reported as the cause of CTS [3–5]. In the case of gout, the masses consist of uric acid crystals, and the resulting elevated levels of leucocytes and uric acid in the blood are useful in making a diagnosis [5]. In the case of pseudogout, the masses consist of calcium pyrophosphate and the patients usually have a history of acute inflammation with diffuse calcification on plain radiography [4]. In the present case, the main composition of the calcareous lesion was calcium phosphate, and there was no sign of acute inflammation, which excluded gout or pseudogout as causes.

In 1967, Harkess and Peters defined the following conditions for diagnosing tumoral calcinosis: (1) the presence of a large, painless, calcified mass around articular sites; (2) the absence of abnormal values of serum calcium or phosphorus; (3) no association with renal diseases, metabolic disorders, or collagen diseases; (4) manifestation of the disease before the age of 20; (5) evidence of familial or racial predisposition; and (6) recurrence of the lesion, particularly after incomplete excision [11]. Although tumoral calcinosis is a known cause of secondary CTS, the term “tumoral calcinosis” itself is controversial [2]. Currently, any calcium-deposit-like tumor around a joint is considered tumoral calcinosis regardless of the patient's age, sex, or preexisting disease [12]. Tumoral calcinosis is subdivided into two types: a primary type without disease association or a secondary type that is associated with other disorders such as chronic renal failure, hyperparathyroidism, malignancy, sarcoidosis, scleroderma, and hypervitaminosis D [2]. Primary tumoral calcinosis without hyperphosphatemia is the most common clinical subtype [13], but approximately 30% of cases are familial and associated with hyperphosphatemia due to the mutation of a gene that encodes fibroblast growth factor-23 or N-acetyl-galactosaminyl transferase enzyme [2, 14]. In the present case, round and calcified masses without inflammation were observed in several parts of patient's body and the patient had no family history or preexisting disease. Therefore, we believe this was a case of CTS caused by nonfamilial, systemic primary tumoral calcinosis. Since incomplete resection can lead to recurrence of lesions [2], a follow-up radiography examination is needed if symptoms recur.
